# Polypeptides Isolated from *Lactococcus lactis* Alleviates Lipopolysaccharide (LPS)-Induced Inflammation in *Ctenopharyngodon idella*

**DOI:** 10.3390/ijms23126733

**Published:** 2022-06-16

**Authors:** Pei Li, Youqing Xu, Yupo Cao, Zhaokun Ding

**Affiliations:** 1 College of Life Science and Technology, Guangxi University, Nanning 530004, China; dalipei@126.com; 2 Institute for Fishery Sciences, Guangxi University, Nanning 530004, China; 3 Agricultural Products Processing Research Institute, Chinese Academy of Tropical Agricultural Sciences, Zhanjiang 524000, China; yupo53@163.com

**Keywords:** polypeptide, *Lactococcus lactis*, *Ctenopharyngodon idella*, lipopolysaccharide, bioassay-guided isolation, anti-inflammatory

## Abstract

The main purpose of the present study was to evaluate the anti-inflammatory activity of *Lactococcus lactis* BL52 and isolate active substances responsible for anti-inflammatory activity. Head-kidney (HK) macrophages were used for in vitro bioassay-guided isolation, and the structure of the two peptides was identified by mass spectrometry analysis. Lipopolysaccharide (LPS)-induced inflammatory responses in *Ctenopharyngodon idella* were also examined to evaluate the in vivo anti-inflammatory activity of active substances. Two active peptides were isolated by HPLC from *L. lactis* BL52, and an in vitro anti-inflammatory assay demonstrated that peptide ALBL1 and ALBL2 dose-dependently inhibited LPS-induced inflammatory cytokines TNF-α, IL-6, and IL-1β and inflammatory factors NO and PGE 2 production in macrophages (*p* < 0.05). After being treated with 20 mg/Kg peptide ALBL1 and ALBL2, the expression levels of TNF-α, IL-6, IL-1β, NO, and PGE 2 were significantly inhibited (*p* < 0.05). Results from the in vivo test showed that when the concentration of peptide ALBL1 and ALBL2 reached 30 mg/Kg, the LPS-induced upregulations of TNF-α, IL-6, IL-1β, NO, and PGE 2 were prevented. In addition, peptide ALBL1 and ALBL2 blocked the expression of Toll-like receptor 2 (TLR2) and then suppressed the phosphorylation of nuclear transcription factor-kappa B (NF-κB) p65 and degradation inhibitor of IκBα. Moreover, *C. idella* treated with peptide ALBL1 and ALBL2 can relieve pathological inflammatory responses caused by LPS. These results suggest that the anti-inflammatory properties of peptide ALBL1 and ALBL2 might be a result from the inhibition of IL-6, IL-1β, and TNF-α expressions through the downregulation of Toll2/NF-κB signaling pathways.

## 1. Introduction

Inflammation is a complex pathological reaction process and plays a very important role in a non-specific immune system. It is a localized protective response against external stimuli such as infection and mechanical injury. The main clinical symptoms of inflammatory reaction are local redness and swelling and partial congestion [1]. Inflammation response mainly includes pro-inflammatory and anti-inflammatory responses, and the imbalance between pro-inflammatory and anti-inflammatory responses can lead to immune damage in the body and even trigger an “inflammatory storm” [2]. Macrophages are the main immune cells in the innate immune system, and their activation plays an important role in the inflammatory reaction of pathogen infection. Macrophages can directly kill pathogens by phagocytosis. It can also indirectly kill pathogens through various pro-inflammatory mediators. Excessive inflammatory mediators produced by activated macrophages are associated with tissue damage, abnormal gill vessels, and intestinal barrier failure.

Lipopolysaccharide (LPS) is a toxic substance synthesized by Gram-negative bacteria (G−) and is a powerful activator of monocytes/macrophages. Macrophages is the main target cell of LPS, and LPS binding with lipopolysaccharide binding protein (LPB) can activate macrophages and then release a variety of growth factors and cytokines that induce inflammation. LPS stimulated macrophages release a variety of pro-inflammatory mediators and cytokines, which may lead to pathological changes and tissue damage [3,4,5]. During chronic inflammation, activated macrophages produce excessive pro-inflammatory mediators, including NO, PGE2, and ROS, as well as pro-inflammatory cytokines, such as TNF-α, IL-1β, and IL-6. All of these substances promote and exacerbate inflammation through synergies with other inflammatory mediators [6,7]. Elevated iNOS expression is usually associated with a variety of inflammatory diseases, including asthma and colitis [8]. cycloxygenase-2 (COX-2) plays a key role in the transformation of arachidonic acid into prostaglandin E2 (PGE 2), and its overexpression is associated with chronic inflammatory diseases, such as arthritis, neurodegenerative diseases, and cancer [9]. Therefore, the regulation of iNOS and COX-2 expressions may be an effective strategy for anti-inflammatory treatments. 

Lactobacillus is a genus of Gram-positive facultative anaerobic or microaerophilic rod-shaped bacteria [10]. More and more research studies have demonstrated that Lactobacillus strains possess significant anti-inflammatory effects. Marzia Sichetti et al. [11] reported the anti-inflammatory effects of a probiotic formulation of *Lactobacillus rhamnosus*, *Bifidobacterium lactis,* and *Bifidobacterium longum* (Serobioma). Kawahara et al. [12] also found that fermented soy milk with Lactococcus lactis subsp. lactis S-SU2 showed great anti-inflammatory properties in in vitro and in vivo tests. Lactobacillus also show anti-inflammatory activities in colitis animal models [13]. In our previous study, we have constructed an in vitro LPS-induced anti-inflammatory pharmacodynamic evaluation model, and *Lactococcus lactis* BL52 showing great anti-inflammatory activity was obtained. The present study was conducted to evaluate the anti-inflammatory activity of *L. lactis* BL52 and isolate active substances responsible for anti-inflammatory activities. 

## 2. Results

### 2.1. In Vitro Anti-Inflammatory Activity of Fraction and Active Peptides from L. lactis BL52

**Figure 1:**
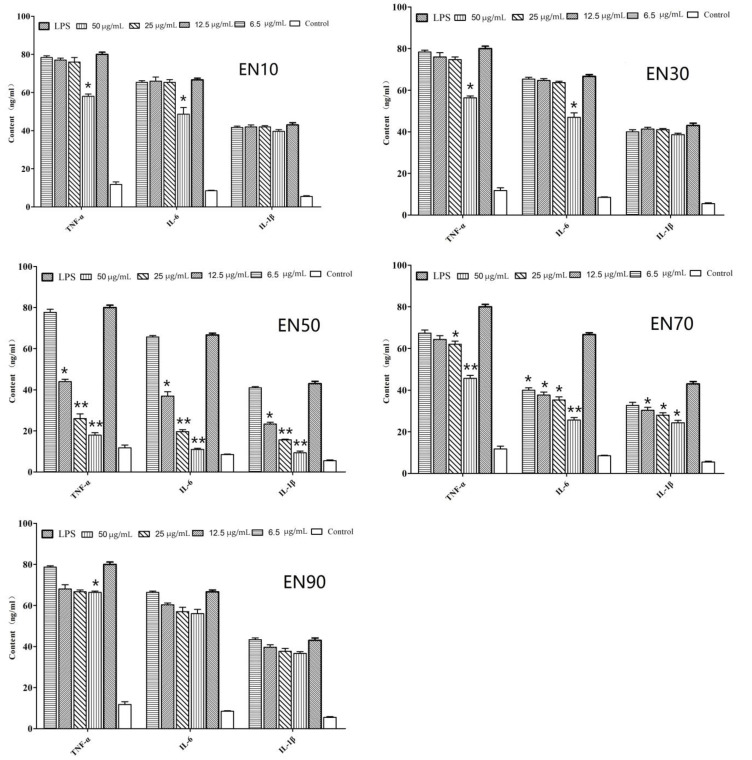
In vitro effect of fractions on the levels of inflammatory cytokine in head-kidney macrophages. Data are means ± SD from three independent experiments performed in triplicate. ** *p* < 0.01; * *p* < 0.05. All the group were only compared with LPS-induced group.

The in vitro anti-inflammatory effects of EN10, EN30, EN50, EN70, and EN90 extracted from *L. lactis* BL52 are shown in Figure 1. The results showed that TNF-α, IL-6, and IL-1β productions of HK macrophages significantly increased after treated with 10 μg/mL LPS for 24 h compared with the untreated group (*p* < 0.01). However, HK macrophages pre-treated with EN50 and EN90 and the production of TNF-α, IL-6, and IL-1β significantly decreased (*p* < 0.05); EN50 showed the highest effect. The production of TNF-α, IL-6, and IL-1β in EN10, EN30, and EN90 were also suppressed; however, most of them showed no difference with the control group (LPS treated). Thus, the EN50 extract was selected for further separation.

**Figure 2:**
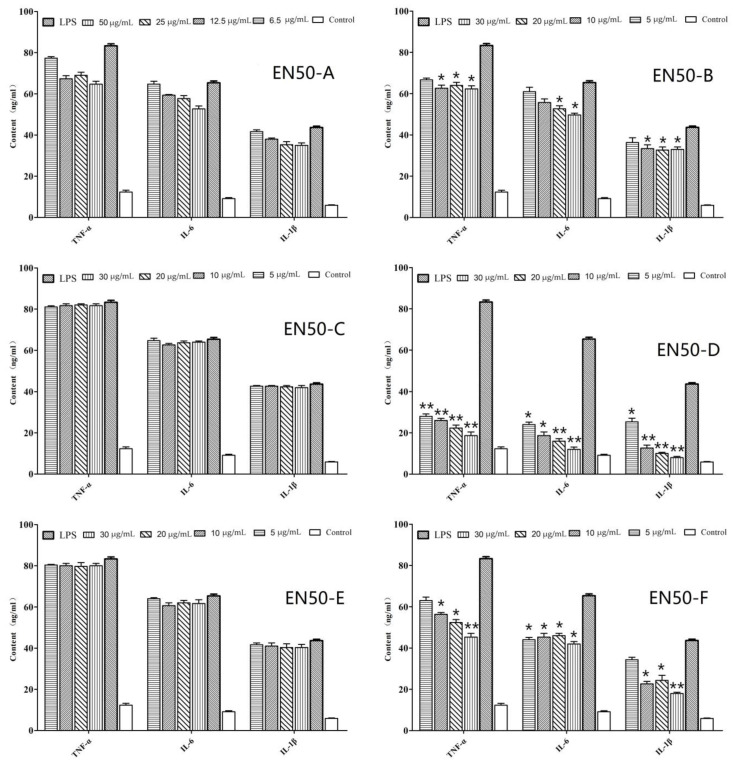
In vitro effect of six fractions from EN50 on the levels of inflammatory cytokine in head-kidney macrophages. Data are means for three assays and presented as the means ± SE. ** *p* < 0.01; * *p* < 0.05. All groups were only compared with the LPS-induced group.

The anti-inflammatory effects of the six fractions (EN50-A, EN50-B, EN50-C, EN50-D, EN50-E, and EN50-F) extracted from EN50 are demonstrated in Figure 2. As shown in Figure 2, EN50-D showed the most anti-inflammatory activity, and the production of TNF-α, IL-6, and IL-1β in the EN50-D-treated group remarkably decreased compared with the LPS-induced group (*p* < 0.01). The production of TNF-α, IL-6, and IL-1β in the EN50-A, EN50-B, and EN50-F groups also were inhibited in a dose-dependent manner. EN50-C and EN50-E showed no anti-inflammatory effects.

**Figure 3:**
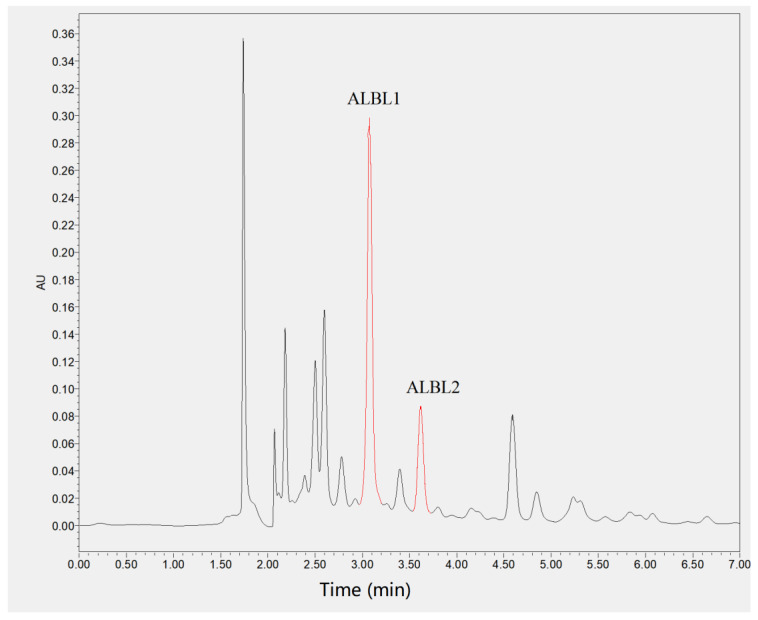
Reverse phase HPLC elution profile of EN50-D from *L. lactis* BL52.

**Figure 4:**
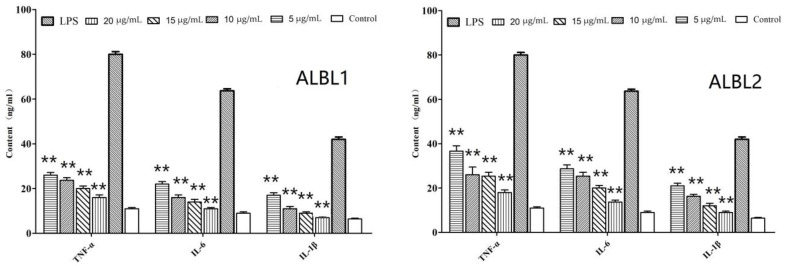
In vitro effect of ALBL1 and ALBL2 on the levels of inflammatory cytokine in head-kidney macrophages. Data are means ± SD from three independent experiments performed in triplicate. ** *p* < 0.01. All groups were only compared with the LPS-induced group.

Ten compounds were isolated and subjected to an in vitro test. Results from the in vitro anti-inflammatory tests showed that two compounds (marked in red in Figure 3 named ALBL1 and ALBL2) showed the greatest anti-inflammatory effects. As for ALBL1, at a concentration of 5 µg/mL, TNF-α, IL-6, and IL-1β in the ALBL1 group were significantly depressed when the concentration reached 20 µg/mL. The contents of TNF-α, IL-6, and IL-1β decreased to the level of the control group (Figure 4). For ALBL2, the in vitro anti-inflammatory effect was slightly lower than ALBL1. When the concentration reached up to 20 µg/mL, only the content of IL-1β decreased to the level of the control group (treated with no LPS and drug).

### 2.2. Mass Spectrometry Analysis

**Figure 5:**
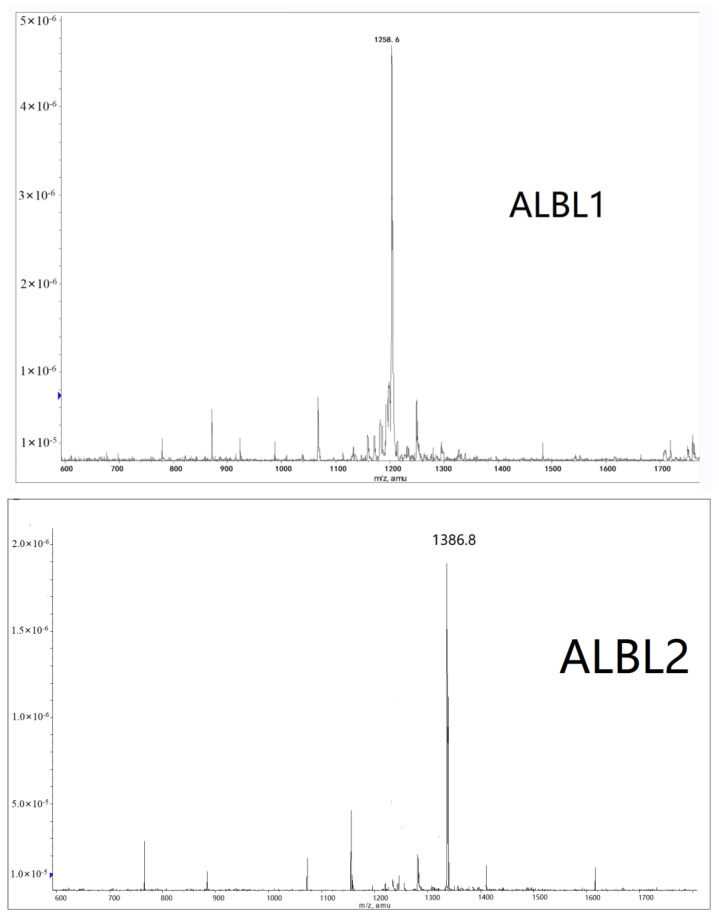
Mass spectrometry analysis of ALBL1 and ALBL2.

Mass spectrometry analyses were conducted for ALBL1 and ALBL2. The results indicated that the molecular masses of ALBL1 and ALBL2 were 1258.6 (M^+1^) and 1386.8 (M^+1^), respectively (Figure 5).

### 2.3. In Vivo Anti-Inflammatory Assay of ALBL1 and ALBL2

#### 2.3.1. In Vivo Effects of ALBL1 and ALBL2 on Cytokine Production

**Figure 6:**
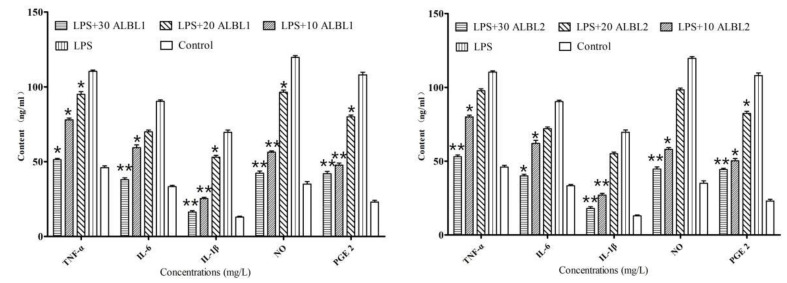
In vivo effect of ALBL1 and ALBL2 on the levels of inflammatory cytokine in *C. idella* after treated with LPS. Data are means ± SD from three independent experiments performed in triplicate. ** *p* < 0.01; * *p* < 0.05. All groups were only compared with the LPS-induced group.

In group II, after being treated with 20 mg/kg LPS, TNF-α, IL-1β, and IL-6 significantly increased compared with group I. However, after being injected with ALBL1, the production of TNF-α, IL-1β, and IL-6 presented a dose-dependent decrease and declined to a base level (group I) when the concentration of ALBL1 was 30 mg/kg (Figure 6).

As for ALBL2, the three cytokine productions (TNF-α, IL-1β, and IL-6) were also suppressed compared with the LPS-induced group (*p* < 0.05) after being treated with 20 mg/kg and 30 mg/kg ALBL1. However, in the low concentration group (10 mg/kg), no differences were observed with the LPS-induced group (Figure 6).

#### 2.3.2. In Vivo Effects of ALBL1 and ALBL2 on the Production of Inflammatory Mediators

An activation in the production of NO and PGE 2 was discovered in the LPS-induced group (Figure 6). However, group III, IV, and V treated with ALBL1 led to a dose-dependent suppression of the production of NO and PGE 2 (*p* < 0.05). In group VI, VII, and VIII, the production of NO and PGE 2 also decreased compared with group II, and it was also significantly higher than group I.

#### 2.3.3. Expression of Toll/NF-KB Signal Pathways Related Genes

**Figure 7:**
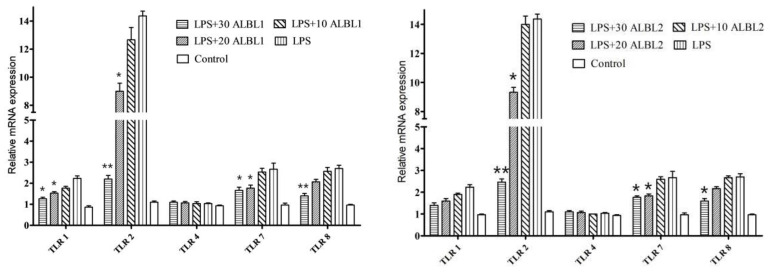
RT-PCR analysis of the expression of TLR related genes expression in *C. idella* after being treated with ALBL1 and ALBL2. Three replicates were set for the tests, with ten fish per replicate. Data are means for three assays and presented as the means ± SE. ** *p* < 0.01; * *p* < 0.05. All groups were only compared with the LPS-induced group.

After LPS treatment, the expressions of TLR1, TLR2, TLR7, and TLR8 in *C. idella* tissues were significantly upregulated. TLR2 was significantly activated, which was 13.2-fold higher than the control group, and it indicated that TLR 2 plays an important role in LPS-induced inflammatory responses. However, after being treated with ALBL1 and ALBL2, TLR2, TLR7, and TLR8 genes were significantly downregulated. There was no significant difference in TLR4 expression between the control group and the LPS-induced group, indicating that LPS did not induce TLR4 expression (Figure 7).

**Figure 8:**
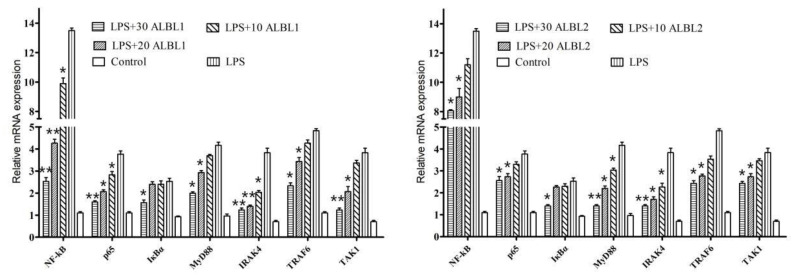
In vivo effect of ALBL1 and ALBL2 on the NF-κB pathway gene expression in LPS-induced *C. idella*. The values presented are mean ± SEM. (*n* = 10 in each group). * *p* < 0.05, ** *p* < 0.01 vs. LPS group (only).

As shown in Figure 8, after being induced by LPS, the levels of IRAK4, TRAF6, p65, TAK1, IκBα, and MyD88 of *C. idella* in kidney were all significantly upregulated compared with the control group. However, after being treated with ALBL1 and ALBL2, the expression levels of IRAK4, TRAF6, p65, TAK1, IκBα, and MyD88 were suppressed compared with the LPS group.

#### 2.3.4. Histological Evaluation

**Figure 9:**
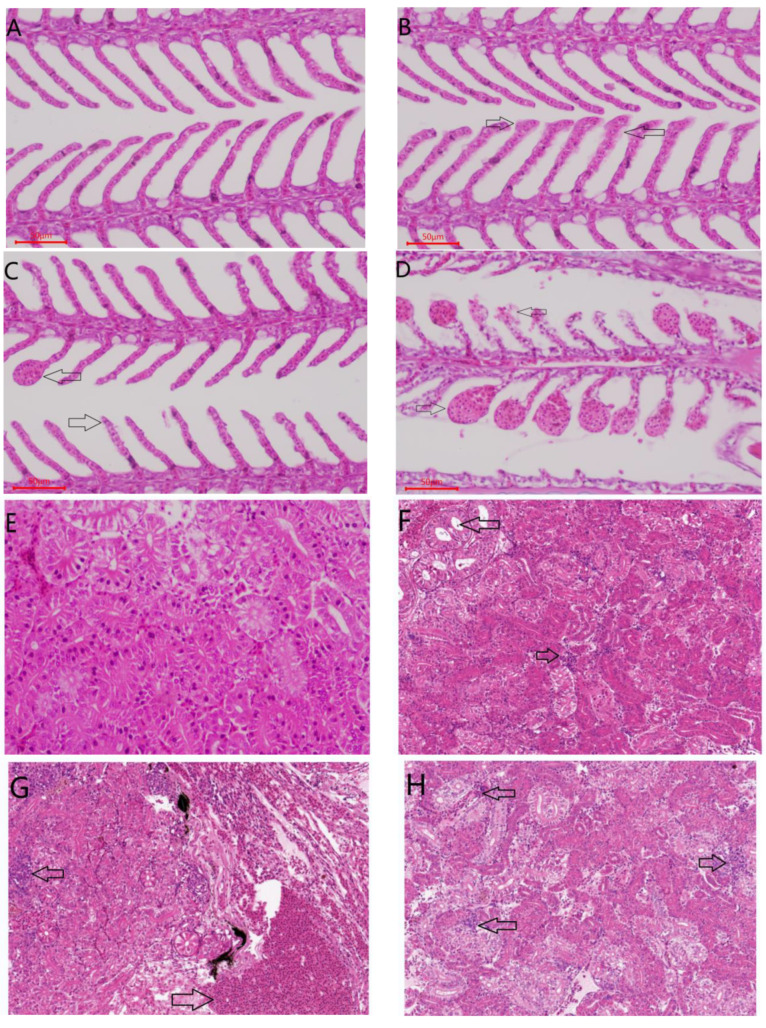

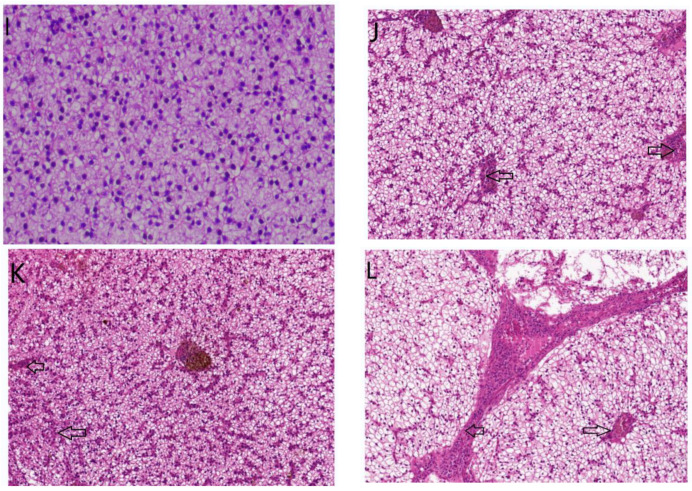
Histopathological assessment of *C. idella* after being treated with ALBL1 and ALBL2. (**A**): Gill in control; (**B**): gill treated with ALBL1; (**C**): gill treated with ALBL2; (**D**): gill treated with LPS; (**E**): kidney in control group; (**F**): kidney treated with ALBL1; (**G**): kidney treated with LPS; (**H**): Kidney treated with ALBL2; (**I**): liver in control group; (**J**): liver treated with ALBL1; (**K**): liver treated with ALBL2; (**L**): liver treated with LPS.

Histopathological results showed that, after being treated with 20 mg/kg LPS, the epithelial cells of gill filaments and lamellae were swollen and degenerated, and the ends of gill lamellae were enlarged to form club-like or spherical structures (Figure 9). After injection with ALBL1 and ALBL2 for 14 days, the pathological symptoms induced by LPS in gill were significantly reduced; however, ALBL1 was more effective than ALBL2.

A large number of inflammatory cell infiltration were found in 20 mg/kg LPS-treated liver, as well as some mild edema of the hepatocytes. After *C. idella* was injected with ALBL1 and ALBL2 for 14 days, the number of inflammatory cell infiltration was reduced compared with the LPS-treated group; the structure of the liver cell in the blank control group was integrated, and the gland was clearly visible (Figure 9).

As compared with the control group, the number of inflammatory infiltrating cells was significantly higher in the LPS-treated group than in the control group, and the structure of kidney cells was intact but with a slight turbid edema. However, after intraperitoneal injections with ALBL1 and ALBL2 for 14 days, the number of inflammatory cell infiltration was also reduced compared with the LPS-treated group; however, pathological symptoms in kidney were less alleviated then in groups treated with ALBL2.

## 3. Discussion

### 3.1. Peptide Inhibited LPS-Induced Inflammatory Cytokines

Inflammation is a pathological process that can cause severe tissue damage in the body. It is a complex and common physiological and pathological process, which can be caused by pathogens such as bacteria and viruses, as well as a series of endotoxins [14]. Endotoxin or bacterial lipopolysacchrides (LPS) can activate monocytes, macrophages, and endothelial cells in body tissues, resulting in the synthesis and release of various cytokines and resulting in a series of inflammatory reactions in the body. Macrophages actively participate in inflammatory responses by releasing pro-inflammatory cytokines: TNF-α, IL-1β, and IL-6 as well as inflammatory cytokines such as NO and PGE2 and recruit additional immune cells to the site of infection or tissue injury [15]. A large number of studies have shown that the concentration of inflammatory cytokines produced by LPS stimulation is a reliable indicator of the degree of inflammation [16]. Thus, the inhibitors of these inflammatory molecules are considered as candidates for anti-inflammatory drugs [8]. Therefore, the present study uses these indicators as evaluation indicators to screen anti-inflammatory drugs. The drug screening mode is the key point for obtaining new and efficient drugs, and an in vitro model has the advantages of convenient operation, short experimental period, etc. Creating an in vivo model is time-consuming compared with an in vitro model, but it can reflect the real situation. Therefore, in the present study, head-kidney (HK) macrophages (LPS-induced) were used for the in vitro bioassay-guided isolation to separate active peptides from *L. lactis* BL52 responsible for anti-inflammatory activities. Two active peptides were isolated and identified by LCMS. These results confirmed that our in vitro anti-inflammatory screening mode was effective and reliable.

Cytokines are a group of glycoproteins with relatively low molecular weight that regulate cellular response and are secreted by a variety of inflammatory cells in the body. They are not only needed for stimulating the responses of the body but they also are the pathological basis for the occurrence and development of tissue injury caused by excessive secretion. Cytokines can not only upregulate the immune function of the body but they also downregulate the defense ability of the body and inhibit inflammatory effects [6]. Macrophages play an important role in inflammation and immune responses. During chronic inflammation, activated macrophages produce excessive pro-inflammatory mediators, including NO, PGE2, and ROS, as well as pro-inflammatory cytokines, such as TNF-α, IL-1β, and IL-6. All these substances promote and exacerbate inflammation through synergies with other inflammatory mediators [6,7]. Elevated iNOS expression is usually associated with a variety of inflammatory diseases, including asthma and colitis [17]. Extensive evidence suggests that high levels or excess production of NO generated by iNOS may result in tissue injuries, vascular abnormalities in gill, gut barrier failure, neurotoxicity, and lipids oxidation [18,19,20,21,22]. In vivo results from our study showed that *C. idella* treated with LPS NO production was significantly increased, but after being with the two peptides, NO production effectively decreased and recovered to a normal level. Cox-2 plays a key role in the transformation of arachidonic acid into prostaglandin E2 (PGE 2), and its overexpression is associated with chronic inflammatory diseases, such as arthritis, neurodegenerative diseases, and cancer [9]. Therefore, the regulation of iNOS and COX-2 expression may be an effective strategy for anti-inflammatory treatment [23,24]. In the present study, the two isolated peptides significantly inhibited the LPS-induced upregulation of PGE 2 by reducing COX-2 expression. The above results suggested that the two isolated peptides could inhibit the expression of NO and PGE 2 production via suppressing the production of iNOS and COX-2.

### 3.2. Peptide Inhibited Downregulated the Toll2/NF-κB Signaling Pathways

Numerous studies have been proved that LPS can bind to toll-like receptors (TLRs) to initiate the intracellular signaling [16]. In mammals, TLR4, as the primary LPS-recognizing receptor, plays an important role in inflammatory responses. MyD88 is considered as an adaptor protein involved in TLRs regulation [23]. MyD88 can recruit downstream signaling molecules such as IRAK4, and the deletion of MyD88 can eliminate responses to LPS stimulation [24,25]. In this study, we detected the expression levels of five TLRs, IRAK4, and MyD88 in vivo. The results showed that only the TLR2 of the five TLRs was induced by LPS, and the content of TLR2 decreased when treated with the two isolated peptides. This may be due to the fact that TLR4 in teleost fish cannot recognize LPS as well as TLR4 in mammals [26]. In order to study the downstream of TLR2, an LPS challenge experiment was conducted in vivo. The results showed that the mRNA expressions of MyD88, IRAK4, and TRAF6 were inhibited after being injected with the two isolated peptides, indicating that the two peptides could eliminate the LPS-induced mRNA expressions of MyD88, IRAK4, and TRAF6 through TLR2.

Transcription factor NF-κB is responsible for many important biological processes in life activities, such as immune response, cell growth, and apoptosis [27,28,29]. It is well known that NF-κB is an important regulator of inflammatory mediators during inflammation, and the expression of multiple cytokine genes, including TNF-α, IL-1β, and IL-6, is associated with NF-κB activation [30]. Therefore, the regulation of NF-κB activation is considered to be a good method to control inflammatory responses. In this study, we found that the two isolated peptides inhibited LPS-induced NF-κB activation of *C. idella*, suggesting that the two isolated peptides mitigated LPS-induced inflammation. To further explore the potential molecular mechanism by which the two isolated peptides inhibit pro-inflammatory cytokine production, we examined the activation of the NF-κB P65 subunit and IκBα in the NF-κB signaling pathway. Our results showed that the phosphorylation of IκBα and (NF-κB) P65 increased in response to LPS, while the two isolated peptides inhibited p65 activation and IκBα degradation. P65 is known to regulate the expression of TNF-α, IL-1β, IL-6, IL-8, β -defensin, iNOS, and IFN-β, which explains the regulation of the two isolated peptides on the expression of TNF-α, IL-1β, IL-6, iNOS, and COX-2 in vivo and in vitro [31]. In conclusion, the anti-inflammatory effects of the two isolated peptides are related to the inhibition of NF-κB activation.

Histopathological results showed that the pathological symptoms induced by LPS in gill and liver were significantly reduced after being treated with polypeptides ALBL1 and ALBL2; however, pathological symptoms in the kidney were less alleviated than in the gill and liver, especially LBL2, which had a poor ability to alleviate inflammatory symptoms in kidneys. This may due to the fact that the absorption and elimination of LPS vary in different organizations. Chen et al. [32] discovered that LPS (from *Cytophaga columnaris*) entered the gill, liver, and other tissues within 3 h after intraperitoneal injected in crucian carp and it almost disappeared in the gill and liver within 48 h; however, it existed in kidney tissues during the test. However, Chen et al. [33] demonstrated that LPS from *Vibrio parahemolyticus* was eliminated faster in liver and kidney tissues than in spleen after injection in *Epinephelus malabaricus*, which indicated that the absorption and elimination of LPS may be related to the source of LPS and species of fish.

## 4. Material and Methods

### 4.1. Fish

Healthy *Ctenopharyngodon idella* (36.8 ± 6.2 g) were obtained from a local fish farm (Guangzhou, China) and were acclimatized to laboratory conditions in a recirculating aquaculture system for 14 days during which no natural dead fish were found. Five fish were randomly selected for parasite detection (microscopic examination on fins, gills, and body surface mucus). Meanwhile, liver, spleen, and kidney were collected for the detection of *C. idella* hemorrhage virus and *Aeromonas versonii*. During the experiment, all fish were fed twice a day at 8:00 am and 17:00 PM. Water quality conditions were as follows: temperature: 26 ± 1 °C dissolved oxygen > 5 mg/L; nitrites < 0.02 mg/L; ammonia < 0.15 mg/L.

*Lactococcus lactis* BL52 was isolated from the gut of *C. idella* and was served in our laboratory.

### 4.2. In Vitro Study

An in vitro assay was conducted to isolate an active compound from *L. lactis* BL52. LPS-induced inflammatory HK macrophages were conducted following our previous study [14]; 1 × 10^5^ cells of HK macrophages was added into 6-well plates and then incubated with extraction, fractions, or active compounds from the fermentation broth of *L. lactis* BL52. After 2 h incubation, they were then treated with 10 µg/mL LPS for 24 h. A supernatant from each well was collected and the levels of TNF-α, IL-6, and IL-1β were measured according to the manufacturer’s instructions (MyBioSource, San Diego, CA, USA). Nitric oxide (NO) was assayed by according to the Griess assay [14]. PGE 2 synthesis was measured according to manufacturer’s instructions of PGE 2 ELISA kit (Cayman, MI, USA). Each group was treated three times.

### 4.3. Bioactivity-Guided Isolation of Active Compound from L. lactis BL52

The active components from *L. lactis* BL52 were separated by bioactivity-guided isolation (in vitro test guided), and the extracts or fractions with high activity were further separated and no active extracts or fractions were abandoned until the active monomeric compounds were obtained.

*L. lactis* BL52 measuring 10 L was added into a 300 L MRS culture medium and then static cultured for 4 days at 28 °C in a 500 L culture tank. After 5 days, 300 L fermentation broth was collected at 6000 rpm/10 min; the liquid supernatant was then concentrated by a vacuum freeze dryer to a dry state. The dry powder was then subjected to macroporous absorbing resin D101 for further separation and gradient eluted with 10%, 30%, 50%, 70%, and 90% ethanol, yielding 5 fractions (EN10, EN30, EN50, EN70, and EN90). Each fraction was collected and then dried by using vacuum freeze-drying technology. Each dried powder was then subjected to an in vitro assay, and EN50 showed the highest anti-inflammatory compared with the other four fractions. Thus, EN50 was then subjected to a Sephadex G-50 column chromatography and successively eluted with citric acid–sodium dihydrogen phosphate (pH 3.5), yielding six fractions (EN50-A, EN50-B, EN50-C, EN50-D, EN50-E, and EN50-F). All the six fractions were also subjected to an in vitro test, and EN50-D showed the most effective anti-inflammatory response compared with the other five fractions; thus, EN50-D was selected for further separation by an HPLC (Octadecyl Silane-A C18 (5 μm 120&Aring; 10 mm × 250 mm, Sigma-Aldrich, Saint Louis, MI, USA) column at 5.0 mL/min flow rate; 30 °C column temperature, 280 nm); mobile phase: 0–30 min with 10% acetonitrile; 30–60 min with 20% acetonitrile; 60–90 min with 30% acetonitrile; 90–120 min with 40% acetonitrile; 120–150 min with 50% acetonitrile; 150–180 min with 60% acetonitrile. All peak values (>0.2) were collected and subjected to an in vitro test.

### 4.4. Mass Spectrometry Analysis

The protein concentration was determined by the bicinchoninic acid procedure as described by the supplier (Sigma-Aldrich, Saint Louis, MO, USA) with bovine serum albumin as a standard. Mass spectral analyses were performed on a Perkin-Elmer Sciex API 165 mass spectrometer (Perkin-Elmer, Shanghai, China) equipped with an ion spray source (C18 column).

### 4.5. In Vivo Experiment

#### 4.5.1. Experimental Design

In vivo tests were conducted according to our previous study [14]. In our preliminary experiment, we carried out in vivo anti-inflammatory efficacy tests of the two peptides but did not achieve the expected results, which may due to the fact that the peptides were digested in the gut; thus, we conducted an injection test. Briefly, 240 *C. idella* were randomly chosen and placed into 24 cycling-filtered plastic tanks, and each tank had 10 fish. All fish were divided into 8 groups: Group I and group II were set as the control groups, which were fed with a control (basic) diet throughout the feeding trial. Groups II, III, IV, V, VI, VII, and VIII were provided with an intraperitoneal injection with 25.0 mg/kg LPS on day 1, and group I received an equal volume of PBS instead of LPS. On day 3, groups III, IV, and V were injected with 10.0, 20.0, and 30.0 mg/kg ALBL1; groups VI, VII, and VIII were injected with 10.0, 20.0, and 30.0 mg/kg ALBL2, respectively. ALBL1 and ALBL2 were given 2 times: once every other day. Fish in the control group were injected with a corresponding dose of PBS. All fish were fed with commercial feed during the in vivo test. On day 7, all fish in the treated group were collected, and blood was sampled.

#### 4.5.2. ELISA Assays of Inflammation

The kidney tissues were homogenized in cold PBS at a 1/10 (*w*/*v*) ratio and centrifuged at 12,640 rpm for 20 min at 4 °C, and then the supernatants were obtained for TNF-α, IL-1β, and IL-6 assays using a fish-specific ELISA kit, as mentioned in Section 4.2.

#### 4.5.3. Reverse Transcription-Polymerase Chain Reaction (RT-PCR)

RT-PCR was conducted to detect the mRNA expressions of inflammatory-related genes TLR1, TLR2, TLR4, TLR7, TLR8, NF-κB, MyD88, IRAK4, TRAF6, and TAK1 and nucleus p65 and IκBα. Briefly, the kidney tissues were homogenized with PBS on ice. The kidney cells were then collected. The total RNA was isolated from the tissues and cells using TRIzol and reverse transcribed into cDNA. Each transcript was identified using specific forward and reverse primers as per the manufacturer’ instructions (Promega, Madison, WI, USA). GAPDH expression was included as an internal, housekeeping gene control. All PCRs were performed at least three times. The primer was designed according to our previous study [14] (Table 1). Data were analyzed by the stratagene MxPro software 4.10 (stratagene mx3005p, Santa Clara, CA, USA).

#### 4.5.4. Histological Evaluation

On day 14 after the secondly injection of drug, three largemouth bass were randomly selected from each group. The liver, kidney, and gill samples in each group were received, fixed in 10% formalin, embedded in paraffin wax, sectioned by microtome, and then hematoxylin and eosin (H&E) stain were conducted to analyze the histological changes of immunized fish.

### 4.6. Data Analysis

All data in this study were performed using the SPSS 19.0 probit procedure; the homogeneity of the replicates of the samples was checked by using the Mann–Whitney *U* test.

## 5. Conclusions

We isolated two active peptides from *Lactococcus lactis* BL52. *C. idella* treated with Peptide ALBL1 and ALBL2 can relieve inflammatory responses caused by LPS through the downregulation of Toll2/NF-κB signaling pathways.

## Figures and Tables

**Table 1:** Primers used for the analysis of mRNA expression by RT-PCR.

Genes	Forward (5′-3′)	Reverse (5′-3′)
NF-kB	GAAGAAGGATGTGGGAGATG	TGTTGTCGTAGATGGGCTGAG
TLR1	TGTGCCACCGTTTGGATA	TTCAGGGCGAACTTGTCG
TLR2	CTATCAAGTGCTCCTCAAA	CCTCACCCATGTAGTATGT
TLR4	CCACCTATTCATCTTTGCCT	GTCTTCCCCTCTTCCACATC
TLR7	GAGCATACAGTTGAGTAAACGCAC	TCTCCAAGAATATCAGGACGATAA
TLR8	TCACATCGCTTCCAGGTCTC	ACGGTGAAATAATGGGGGTT
NF-κB p65	AACCAAGAACCAGCCATACAAG	AACCAAGAACCAGCCATACAAG
IκB	TCTTGCCATTATTCACGAGG	TGTTACCACAGTCATCCACCA
MyD88	GACTGTCGCCGAAATGA	TGCCTTCTCGCTCCTGT
IRAK4	CTCCACACTGAGAGCTTTATC	ATGTGCAGCTGTGTGTATCT
TRAF6	TCACTCACTGTCAGATGTC	TGTTGGCTCTTGTGTTCA
TAK1	AGACAGGACAGACACCAAT	CATCTTACAGTGCTGCTCAA
GAPDH	AGGGGCTCAGTATGTTGTGG	CTCTCTTGGCACCACCCTTA

## Data Availability

No data reported.
